# Genomic surveillance of SARS-COV-2 reveals diverse circulating variant lineages in Nairobi and Kiambu Counties, Kenya

**DOI:** 10.1186/s12864-022-08853-6

**Published:** 2022-09-01

**Authors:** Josiah O. Kuja, Bernard N. Kanoi, Renzo F. Balboa, Clement Shiluli, Michael Maina, Harrison Waweru, Kimita Gathii, Mary Mungai, Moses Masika, Omu Anzala, Matilu Mwau, Taane G. Clark, John Waitumbi, Jesse Gitaka

**Affiliations:** 1 Mount Kenya University, Thika, Kenya; 2 University of Copenhagen, Copenhagen, Denmark; 3 United States Army Medical Research Directorate, Kisumu, Kenya; 4 Kenya Medical Research Institute, Nairobi, Kenya; 5 University of Nairobi, Nairobi, Kenya; 6 London School of Hygiene & Tropical Medicine, London, UK

**Keywords:** SARS-CoV-2, Variant, Surveillance, Vaccine, Genomics

## Abstract

Genomic surveillance and identification of COVID-19 outbreaks are important in understanding the genetic diversity, phylogeny, and lineages of SARS-CoV-2. Genomic surveillance provides insights into circulating infections, and the robustness and design of vaccines and other infection control approaches. We sequenced 57 SARS-CoV-2 isolates from a Kenyan clinical population, of which 55 passed quality checks using the Ultrafast Sample placement on the Existing tRee (UShER) workflow. Phylo-genome-temporal analyses across two regions in Kenya (Nairobi and Kiambu County) revealed that B.1.1.7 (Alpha; *n* = 32, 56.1%) and B.1 (*n* = 9, 15.8%) were the predominant lineages, exhibiting low Ct values (5–31) suggesting high infectivity, and variant mutations across the two regions. Lineages B.1.617.2, B.1.1, A.23.1, A.2.5.1, B.1.596, A, and B.1.405 were also detected across sampling sites within target populations. The lineages and genetic isolates were traced back to China (A), Costa Rica (A.2.5.1), Europe (B.1, B.1.1, A.23.1), the USA (B.1.405, B.1.596), South Africa (B.1.617.2), and the United Kingdom (B.1.1.7), indicating multiple introduction events. This study represents one of the genomic SARS-CoV-2 epidemiology studies in the Nairobi metropolitan area, and describes the importance of continued surveillance for pandemic control.

**Supplementary Information:**

The online version contains supplementary material available at 10.1186/s12864-022-08853-6.

## Introduction

The COVID-19 pandemic, caused by the severe acute respiratory syndrome coronavirus 2 (SARS-CoV-2) has spread globally. The emergence of novel variants (e.g., Alpha, Delta, and Omicron) affects infection control measures, leading to policy changes in social restrictions, and impacts vaccine efficacy. An understanding of the genetic diversity, phylogeny and lineages of SARS-CoV-2, particularly through genomic sequencing, provides insights into circulating infections, the robustness and design of vaccines, and other infection control measures [1, 2]. To date, there have been more than 11 million reported infections and 239,000 reported deaths caused by the novel coronavirus in Africa [3]. In the early months of the COVID-19 pandemic, Africa’s rapid and coordinated response informed by emerging data led to infection control measures which mitigated effects of a first-wave and to a lesser degree a second-wave [4]. This included rapid response through genomic surveillance to curb the spread of SARS-CoV-2. Nigeria took 3 days to sequence the SARS-CoV-2 genome after the identification of the virus [5]. Within the same period, the Network for Genomic Surveillance in South Africa (NGS-SA) was established to facilitate case confirmation and sequencing of the positive cases for phylogenetic and lineage updates [6]. Public health officers in Uganda also established a program to facilitate genomic sequencing of confirmed positive samples from rapid contact tracing and international arrivals [7]. However, in 2022, as vast vaccination campaigns have enabled the global north to gain some control over the pandemic, the vaccine roll-out in Africa lags because of inequities in access. Kenya has vaccinated 12,652,991 people at a rate of 23.89 doses per 100 people [8].

Kenya joined the genomic surveillance of the SARS-CoV-2 pandemic after reporting the first case on 13th March, 2020 [9]. Earlier cases were dominated by the B.1 lineage, which was introduced into African countries from international arrivals, predominantly of European origin. Early public health measures in Kenya included restricted movement through the limitation of social interaction and gatherings, but failed to prevent transmission [10]. By the end of July 2020, the Kenyan Ministry of Health had reported 20,636 PCR confirmed cases and 341 SARS-CoV-2 associated deaths. Most cases were from Nairobi and Mombasa, which were exposed to cross-border interactions and international arrivals, including individuals who did not undergo rapid testing procedures at border control checkpoints. At the time, seroprevalence surveillance of the national blood bank revealed the existence of SARS-CoV-2 in the population before the 13th of March 2020 [11]. The growing prevalence was confirmed by community-based modelling teams which were able to identify different variants for each wave in the country based solely on the seroprevalence, PCR confirmed cases, and genomic data [12].

Genomic surveillance is an essential approach to characterise the transmission dynamics and the prevalence of SARS-CoV-2 within a population. Most of the sequences published in Kenya have been closely related to the Wuhan reference sequences characterised by between 4 and 16 nucleotide substitutions [13]. The predominant nucleotide substitutions were associated with mutations at positions ﻿A23403G (﻿D614G; S gene), ﻿ P970L (S gene), P314L (ORF1b), R203K (N) and G204R (N) [13]. However, genomic surveillance revealed the D614G spike mutation as the dominant mutation across Kenya and its neighbouring states despite its initial appearance in the earlier stages of the pandemic [14].

Here, we sequenced RT-PCR confirmed SARS-CoV-2 positive samples from Nairobi and Kiambu County. The samples were collected between September 2020 and March 2021, spanning the severe Alpha and Delta variants of concern within Kenya and across borders. This work led to 57 SARS-CoV-2 isolate sequences available for phylogenomic analyses across Nairobi and Kiambu County representing one of the largest genomic epidemiology studies in the Nairobi metropolitan area.

## Methods

### Sample collection

Sample collection and testing were conducted according to the Kenya Ministry of Health (MoH, Kenya) COVID-19 pandemic surveillance protocols and guidelines [15]. Sampling and whole genome sequencing protocols were reviewed and approved by the Ethics Review Committee (ERC-MKU/ERC/1613) of Mount Kenya University. The study was conducted between September 2020 and March 2021, consisting of nasopharyngeal samples collected using nasal swabs. The collected swabs were stored in viral transport media tubes until use. One hundred fifty microliter of each sample was processed for RNA extraction for sequencing.

### SARS-CoV-2 diagnosis and RNA extraction

RNA extraction was performed using the Sacace Biotechnologies Ribo Virus kit protocol (Sacace SARS-CoV-2 Variants Typing Real-TM; Srl-Via Scalabrini, Como, Italy) according to manufacturer’s instructions. Subsequently, positive infections were quickly identified through RNA purification followed by real time reverse-transcription Polymerase Chain Reaction (RT-PCR) using a Ribo virus column (Srl-Via Scalabrini, Como, Italy).

### SARS-CoV-2 genome amplification, library preparation and sequencing

The purified RNA was used to synthesise complementary DNA (cDNA) using random primers with the Superscript IV one step reverse transcriptase kit (Thermo Fisher Scientific, CA, USA). The cDNA was then amplified using the multiplex ARTIC primer-pools A and B version 3 [16] using the NEBNext Q5 High-Fidelity 2X Master Mix (New England Biolabs, MA, USA). The resulting PCR products were pooled together and cleaned using 1× AMPure XP beads (Beckman Coulter), and then used for library preparation with the NexteraXT library preparation kit (Illumina, CA, USA), following the manufacturer’s instructions. The final library was normalised to 12 pM, spiked with 10% Phix genome and sequenced on Illumina MiSeq platform (Illumina, CA, USA) using 600 V3 paired-end chemistry.

### SARS-CoV-2 lineage and clade assignment

Amplicon sequences from 57 RT-PCR positive samples were assembled against SARS CoV-2 reference genomes using the IDseq platform [17]. The resulting sequences were then assigned to SARS-CoV-2 lineages using Pangolin (v2.1.6) [18]. Among the 57 samples, 55 passed quality checks using the Ultrafast Sample placement on the Existing tRee (UShER) workflow for subsequent phylogenetic analyses [19]; two isolates were, however, removed due to an excess of N-bases (> 0.50). Nucleotide mutations and amino acid changes within 12 SARS-CoV-2 genes for all samples were subsequently detected through NextClade (v0.13.0), using standard parameters [20]. Finally, phylogenetic assignments were performed using a maximum likelihood approach in IQ-TREE, using default parameters [21].

## Results

### SARS-CoV-2 testing and clinical characteristics

**Fig. 1:**
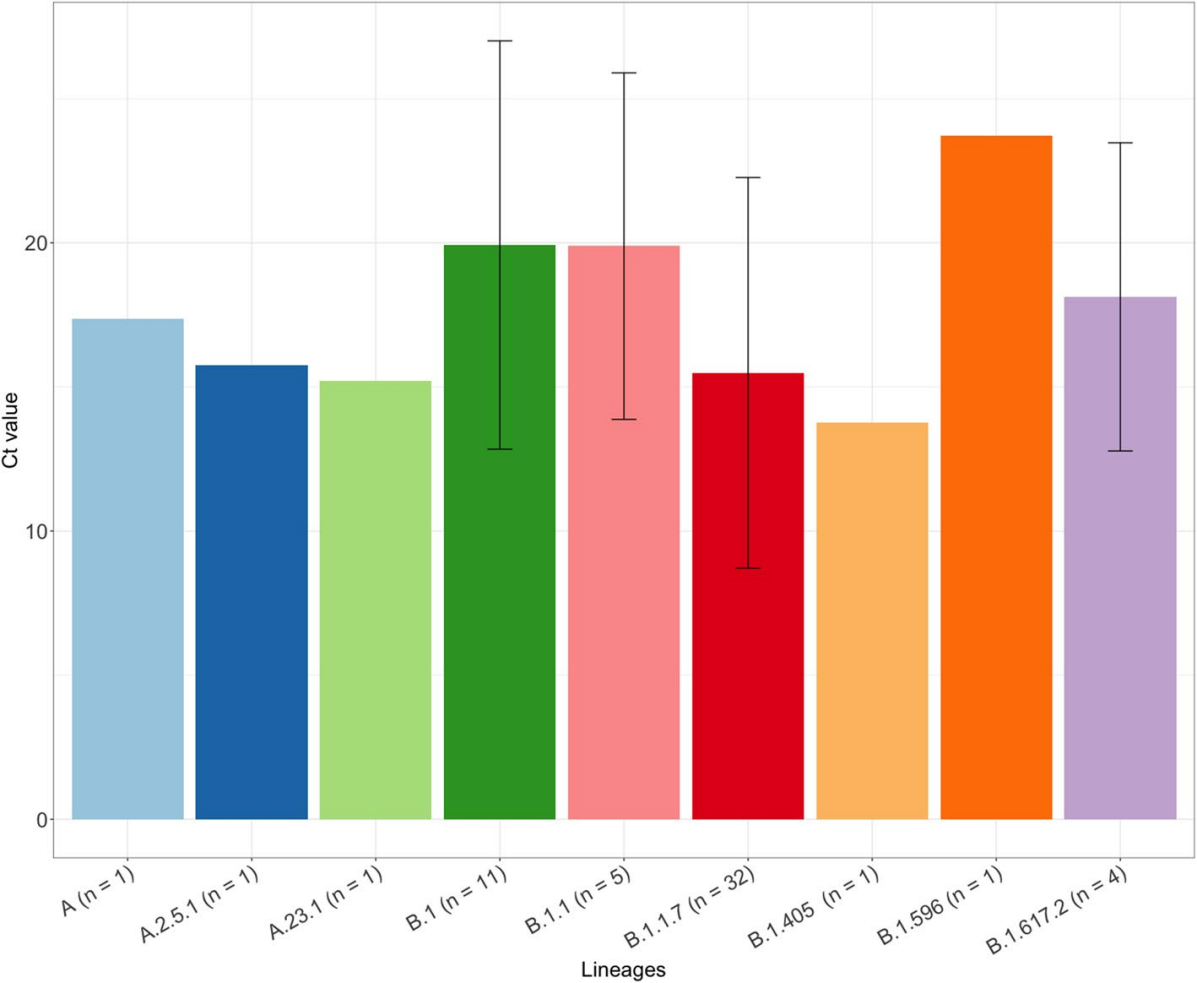
Bar Plots of cycle threshold (Ct) values in different variants. n: number of samples related to the variant in the Pangolin lineage

**Fig. 2:**
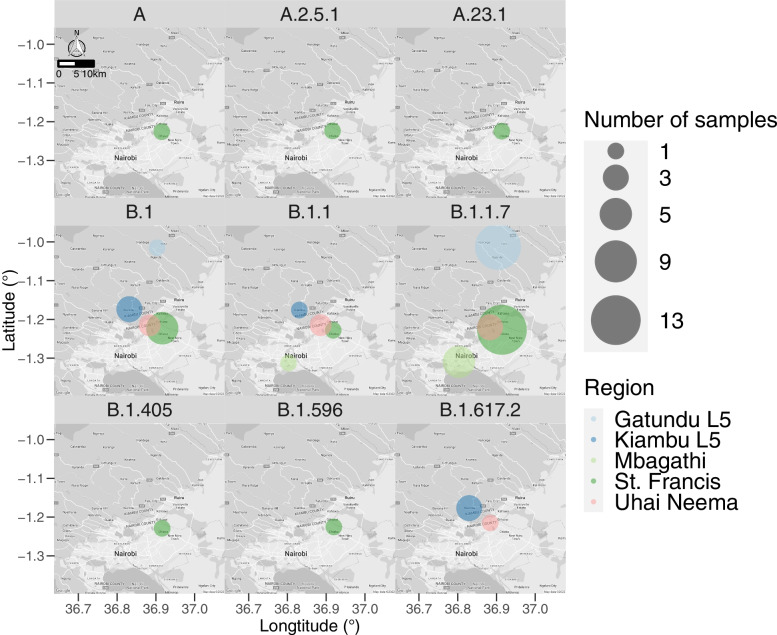
Number of RT-PCR positive samples per lineage. Bubble sizes correspond to the number of RT-PCR positive samples for each lineage from five collection sites: Gatundu (furthest north), Kiambu (center), Uhai Neema (central east), St. Francis (furthest east), and Mbagadhi (furthest south). Maps were generated in RStudio (v4.1.3) using the ggmap library on 3/08/2022 with the WGS84 coordinate reference system [23]

During the study period, SARS-CoV-2 RT-PCR testing identified 57 positive samples from the five COVID-19 testing sites in Nairobi and Kiambu County. Specifically, 38 samples were obtained from Nairobi (St. Francis Community Hospital, *n* = 24; Uhai Neema Hospital, *n* = 8; and Mbagathi Hospital, *n* = 6) and 19 from Kiambu County (Gatundu Level 5 Hospital, *n* = 12; Kiambu Level 5 Hospital, *n* = 7) (Table S1). The observed Ct values, a measure of relative abundance of virus material in a sample [22], from the positive samples ranged between 3.96–31.59 with a total of 26 asymptomatic and 31 symptomatic patients. Out of the 26 asymptomatic patients, 12 (46.2%) were males while 14 (53.8%) were female. For the 31 symptomatic patients, 20 (64.5%) were males and 11 (35.5%) were females. The mean of the Ct values of the samples varied between Nairobi and Kiambu counties (Fig. 1). Variation was also associated with the lineage variants (Figs. 1 and 2).

### Inferred lineages

**Fig. 3:**
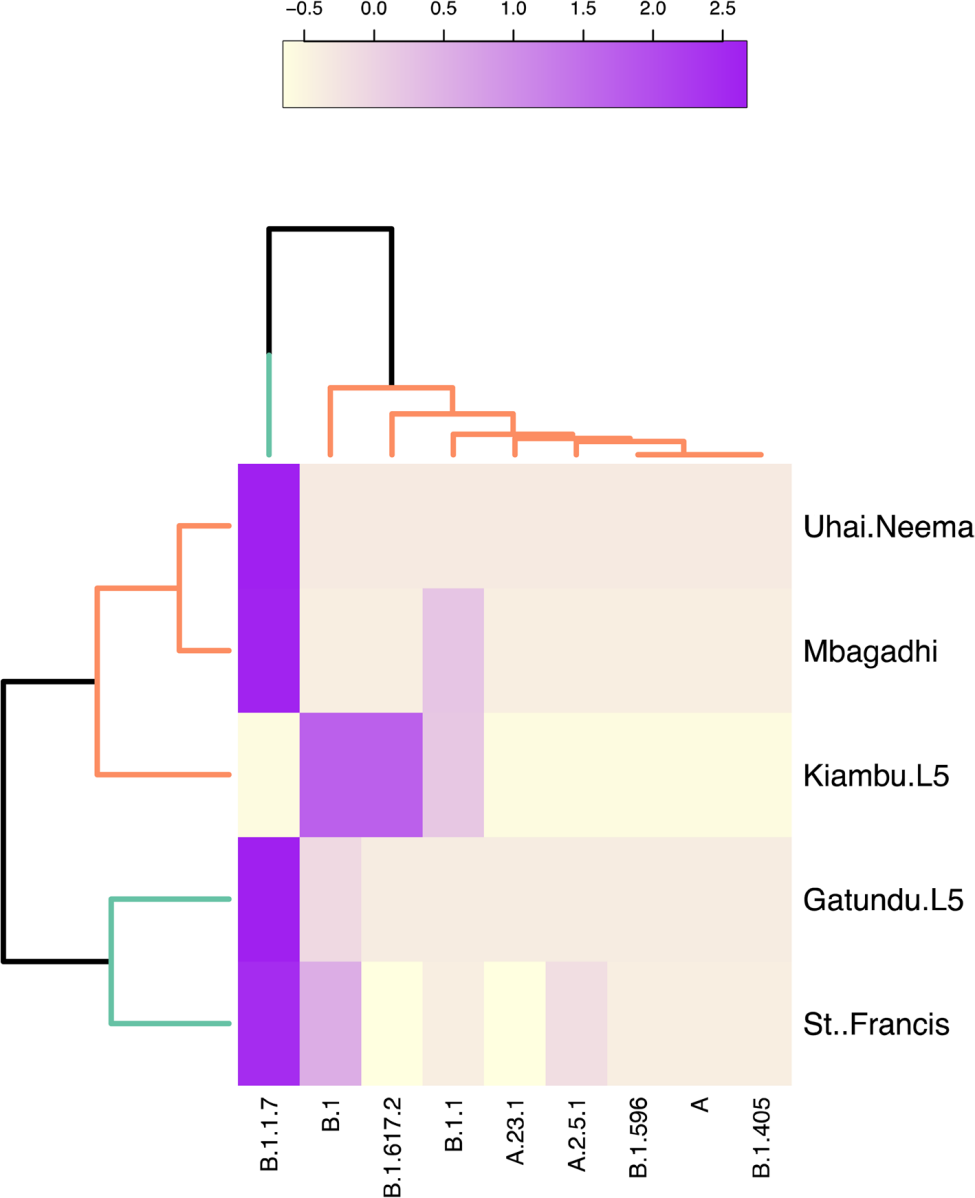
Hierarchical clustering of the identified lineages across Nairobi and Kiambu County. The colour scale represents the frequency of occurrence of specific lineages within a specific site in a scale of − 0.5 – 2.5

Sequence analyses of the 57 isolates (*n* < 0.50) revealed 9 major SARS-CoV-2 lineage variants originating from major hotspots across the globe. These include China (A), Costa Rica (A.2.5.1), Europe (A.23.1, B.1, B.1.1), the USA (B.1.405, B.1.596), South Africa (B.1.617.2), and the United Kingdom (Alpha; B1.1.7), indicating a significant rate of transmission across borders into Kenya. B.1.1.7 was the most prevalent lineage detected in all samples (Alpha; *n* = 32, 56.1%), followed by B.1 (*n* = 11; 19.2%), B.1.1 (*n* = 5; 8.8%), B.1.617.2 (*n* = 4; 7.0%), A.2.5.1 (*n* = 2; 3.5%), A, A.23.1, B.1.405 and B.1.596 (n = 1; 1.8% respectively) (Fig. 2). In St. Francis Hospital (*n* = 24), B.1.1.7 (*n* = 13; 54.2%) and B.1 (*n* = 5; 20.8%) were dominant, followed by A, A.2.5.1, A.23.1, B.1.1, B.1.405, and B.1.596 (*n* = 1, respectively). Mbagathi samples exhibited two lineages, B.1.1.7 (*n* = 5) and B.1.1 (*n* = 1). B.1 (*n* = 3), B.1.617.2 (*n* = 3), and B.1.1 (*n* = 1) lineages dominated Kiambu Level 5 Hospital. Gatundu Level 5 Hospital was dominated by B.1.1.7 (*n* = 11; 91.7%), with B.1 (*n* = 1; 8.3%) present. Finally, B.1.1.7 (*n* = 3), B.1.1 (*n* = 2), B.1 (*n* = 2) and B.1.617.2 (*n* = 1) were detected at Uhai Neema Hospital in Nairobi County (Figs. 2 and 3).

### SARS-CoV-2 sequence diversity

**Fig. 4:**
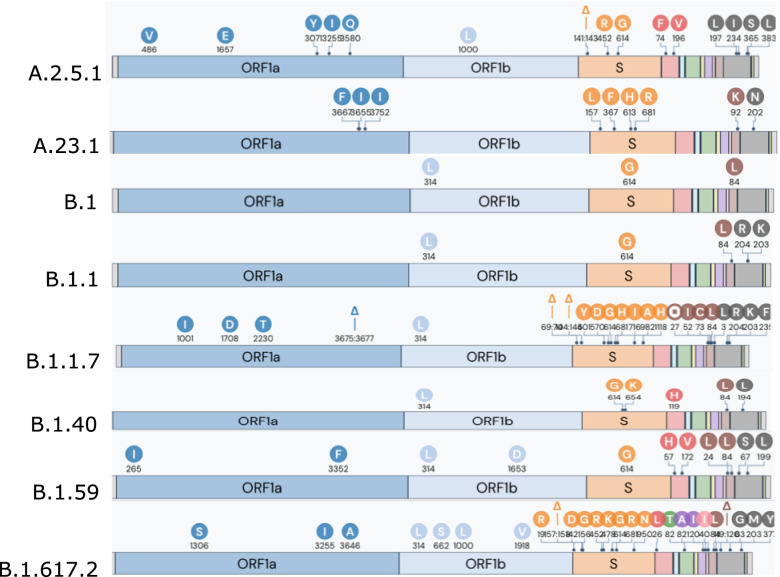
Genetic profile of the identified variants and the corresponding gene variations for characteristic lineage mutation

**Fig. 5:**
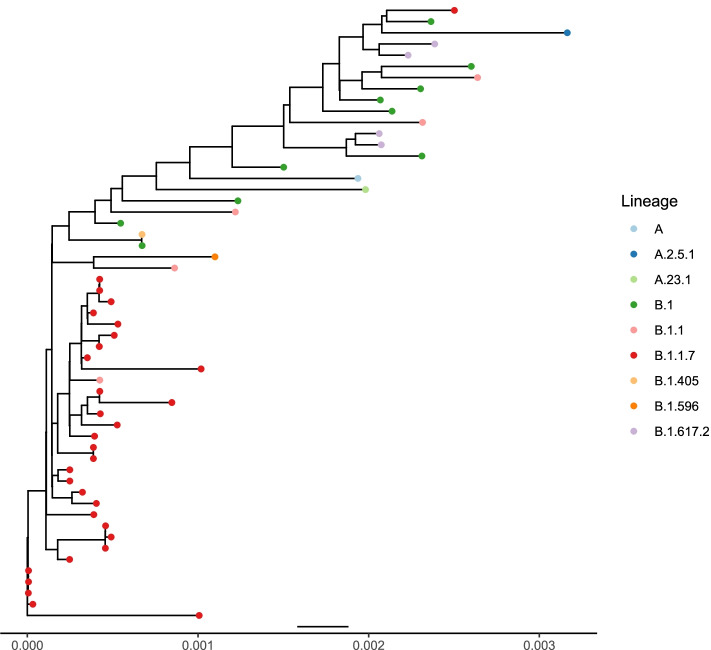
Mutational resolved tree describing sample variation. The tip colour represents samples collected from each of the healthcare units. The x axis represents the lineage genetic diversity among the samples

UShER quality checks skipped two isolates due to an excess of N-bases (> 0.50), yielding a total of 55 sequences for phylogenetic analyses. Sequence variants across the 55 isolates using NextClade described nucleotide mutations (range: 5 to 48) leading to 4 amino acid changes (range: 4 to 23) and deletions (range: 2 to 8). Mutations were found across 12 genes, with the greatest number in samples associated with B.1.1.7. As expected, 12 genes were identified with a size range of 5–4000 base pairs (Fig. 4) [24]. Most genes exhibited similar sequences as indicated by the clade assignments, mutation calls and sequence quality checks. The S gene, which codes for the spike protein, and ORF1a were the most diverse genes across lineages (Fig. 4). Phylogenetic analyses revealed the prevalence and emergence of lineages in Nairobi and Kiambu counties. Lineages were clustered based on their specific variants indicating B.1.1.7 as the predominant lineage in the phylogeny despite the mutational variation and temporal differences within isolates (Fig. 5).

## Discussion

The study revealed the B.1.1.7 lineage as the predominant variant in Nairobi and Kiambu County (two neighbouring counties with high populations). The global cumulative prevalence of the B.1.1.7 lineage is 19% [25], similar to its prevalence in Kenya, which is estimated to be between 10 and 20% with a total of 947 positive samples out of 5175 sequences [25] at the time of sampling. Despite the emergence of the Delta variant (B1.617.2) at the time of sample collection, the dominance of the B.1.1.7 lineage in Kenya, particularly within the capital city, suggests that Delta was mostly locally transmitted. Ct values above 30 are an indication of a reduced concentration of viral particles within an individual, low infectivity, and are associated with patients that would not require isolation and quarantine, especially if the symptoms appeared 10 days before the RT-PCR test [26]. In this study, the mean Ct values were mostly lower than 25, particularly in isolates exhibiting the B.1.1.7 lineage (Fig. 1), suggesting that these patients were highly infectious. They would be referred to as super spreaders if the viral preparations could be calibrated to 1 million copies of SARS-CoV-2 RNA per ml [27, 28], and thus would require isolation to contain the transmission of the virus within the population.

B.1 was the second most abundant (19.2%) lineage in Nairobi and Kiambu. In Nairobi County, it was detected in St. Francis Community Hospital with a relative abundance of 20.8%. The variation in lineage distribution between the two counties is an indication of local transmission through a geographical timescale. Globally, 102,145 sequences of B.1 lineage have been identified with a cumulative frequency of 25% [25]. Since its first identification in the United States of America, the variant has spread across countries including Kenya with a cumulative prevalence of 20–35%. B.1 was reported to be the predominant lineage at the Kenyan Coast, Mombasa County [13]. Most of the cases at the Kenyan coast were from international arrivals and travellers. By then Mombasa County was experiencing high death rates with exponential positivity rates unlike any other county in Kenya. Lineages identified in our study population must have been localised transmissions after a wave of the B.1 at the coastal region. The rest of the lineages were homogeneously distributed across the clinical units. Their occurrence was, however, specific to healthcare facilities indicating the success of local transmissions across the counties. Apart from the B.1 and A lineages that were detected at the Kenyan coastal region [13], the rest of the lineages in our study were specific to Nairobi and Kiambu County. Even the abundant B.1.1.7 Alpha lineage was never detected at the Kenyan coast and its borders at the time of the B.1 outbreak in the region [13].

Dynamics of the identified lineages in the population across the two counties could be due to many factors, including socio-economic parameters. The genomic epidemiology of the variants, however, underpins the epidemic waves across Africa and Kenya. Nairobi is a cosmopolitan city with diverse interactions from international travellers. Kiambu is an equally busy county in which populations interact through trade and travels. These two counties are also characterised by the high number of students in the population [29], hence the variation between sub-populations in these two counties could be significant and are likely to determine the outcome of SARS-CoV-2 variants prevalence. Ostensibly, sub-groups of lower socioeconomic status are more likely to encounter SARS-CoV-2 variants compared with those from higher socioeconomic groups [12]. As expected, the Omicron variant (B.1.1.529) was not detected in this study. This agrees with the currently earliest detection of Omicron at the end of 2021, which postdates the sample collection period [30]. Though isolates in this study do not show any relations to the Omicron (B.1.1.529) variant of concern, the variant is likely to spread in Nairobi and Kiambu counties due to high rates of mutation from the previous variants (Delta) and super spreading between populations. It would occur with symptoms like those of the previous variants with less severe infections. The severity of Omicron and any other emerging COVID-19 variant infection can, however, be prevented by vaccination as the best public health measure protecting people from severe illnesses.

In conclusion, the SARS-CoV-2 lineages and genetic isolates identified in this study could be traced back to multiple countries including China (A), Costa Rica (A.2.5.1), Europe (B.1, B.1.1, A.23.1), the USA (B.1.405, B.1.596), and South Africa (B.1.617.2), and the United Kingdom (Alpha;B1.1.7), indicating a significant rate of transmission across borders into Kenya. Using the established platforms, continued surveillance will be required to give a deeper understanding of the spread of SARS-CoV-2 and detect any emerging variants that may be of interest to support pandemic control.

## Supplementary Information

**Additional file 1: Table S1.** Isolate metadata.

## Data Availability

The datasets generated and analysed for the study are available at GISAID (GISAID identifier: EPI_SET_20220725gk; doi: 10.55876/gis8.220725gk). All genome sequences and associated metadata in this dataset are published in GISAID’s EpiCoV database. To view the contributors of each individual sequence with details such as accession number, Virus name, Collection date, Originating Lab and Submitting Lab and the list of Authors, visit 10.55876/gis8.220725gk.
