# Supplementary material for: Genomic surveillance of SARS-COV-2 reveals diverse circulating variant lineages in Nairobi and Kiambu Counties, Kenya
Source: BMC Genomics. 2022 Sep 1;23:627. doi: 10.1186/s12864-022-08853-6 (PMC9434529; doi:10.1186/s12864-022-08853-6)
Supplement: Supplementary file 1 — Additional file 1: Table S1. Isolate metadata. [file 12864_2022_8853_MOESM1_ESM.docx]

Table S1. Isolate metadata

| Isolate | Lineage | Source | Clinical status | CT Value | Sample type | Amount | Region |
| --- | --- | --- | --- | --- | --- | --- | --- |
| MKU_1 | B.1.1.7 | Female | SYMPTOMATIC | 18.7 | Nasopharyngeal | 50 μL | St. Francis |
| MKU_2 | B.1 | Female | SYMPTOMATIC | 18.94 | Nasopharyngeal | 50 μL | St. Francis |
| MKU_4 | B.1.1.7 | Male | SYMPTOMATIC | 16.88 | Nasopharyngeal | 50 μL | St. Francis |
| MKU_6 | B.1 | Male | ASYMPTOMATIC | 12.22 | Nasopharyngeal | 50 μL | St. Francis |
| MKU_7 | B.1.1.7 | Female | SYMPTOMATIC | 12.3 | Nasopharyngeal | 50 μL | St. Francis |
| MKU_8 | B.1.1.7 | Female | SYMPTOMATIC | 12.96 | Nasopharyngeal | 50 μL | St. Francis |
| MKU_9 | B.1.405 | Female | SYMPTOMATIC | 13.77 | Nasopharyngeal | 50 μL | St. Francis |
| MKU_10 | B.1.1.7 | Male | SYMPTOMATIC | 13.81 | Nasopharyngeal | 50 μL | St. Francis |
| MKU_11 | B.1.1 | Female | SYMPTOMATIC | 23.71 | Nasopharyngeal | 50 μL | St. Francis |
| MKU_12 | B.1.1.7 | Male | SYMPTOMATIC | 15.65 | Nasopharyngeal | 50 μL | St. Francis |
| MKU_13 | A.2.5.1 | Male | SYMPTOMATIC | 15.21 | Nasopharyngeal | 50 μL | St. Francis |
| MKU_14 | B.1.1.7 | Male | ASYMPTOMATIC | 15.79 | Nasopharyngeal | 50 μL | St. Francis |
| MKU_15 | B.1.1.7 | Male | ASYMPTOMATIC | 6.79 | Nasopharyngeal | 50 μL | St. Francis |
| MKU_16 | B.1 | Male | SYMPTOMATIC | 19.72 | Nasopharyngeal | 50 μL | St. Francis |
| MKU_17 | A | Female | ASYMPTOMATIC | 17.37 | Nasopharyngeal | 50 μL | St. Francis |
| MKU_18 | B.1.1.7 | Male | ASYMPTOMATIC | 9.47 | Nasopharyngeal | 50 μL | St. Francis |
| MKU_19 | B.1 | Male | SYMPTOMATIC | 24.25 | Nasopharyngeal | 50 μL | St. Francis |
| MKU_20 | B.1.1.7 | Male | SYMPTOMATIC | 22.14 | Nasopharyngeal | 50 μL | Mbagadhi |
| MKU_21 | B.1.1 | Male | ASYMPTOMATIC | 15.74 | Nasopharyngeal | 50 μL | Mbagadhi |
| MKU_22 | B.1.1.7 | Male | SYMPTOMATIC | 5.84 | Nasopharyngeal | 50 μL | Mbagadhi |
| MKU_23 | B.1.1.7 | Male | SYMPTOMATIC | 8.89 | Nasopharyngeal | 50 μL | Mbagadhi |
| MKU_24 | B.1.1.7 | Male | SYMPTOMATIC | 16.66 | Nasopharyngeal | 50 μL | Mbagadhi |
| MKU_25 | B.1.1.7 | Male | SYMPTOMATIC | 24.98 | Nasopharyngeal | 50 μL | Mbagadhi |
| MKU_27 | B.1.1.7 | Female | ASYMPTOMATIC | 11.76 | Nasopharyngeal | 50 μL | Gatundu L5 |
| MKU_29 | B.1.1.7 | Female | SYMPTOMATIC | 25.92 | Nasopharyngeal | 50 μL | Gatundu L5 |
| MKU_30 | B.1.1.7 | Female | ASYMPTOMATIC | 3.96 | Nasopharyngeal | 50 μL | Gatundu L5 |
| MKU_31 | B.1.1.7 | Male | ASYMPTOMATIC | 30.96 | Nasopharyngeal | 50 μL | Gatundu L5 |
| MKU_32 | B.1.1.7 | Female | SYMPTOMATIC | 14.89 | Nasopharyngeal | 50 μL | Gatundu L5 |
| MKU_33 | B.1.1.7 | Female | ASYMPTOMATIC | 22.03 | Nasopharyngeal | 50 μL | Gatundu L5 |
| MKU_34 | B.1.1.7 | Female | ASYMPTOMATIC | 11.2 | Nasopharyngeal | 50 μL | Gatundu L5 |
| MKU_35 | B.1.1.7 | Male | SYMPTOMATIC | 15.21 | Nasopharyngeal | 50 μL | St. Francis |
| MKU_36 | B.1.1.7 | Female | ASYMPTOMATIC | 9.55 | Nasopharyngeal | 50 μL | Gatundu L5 |
| MKU_37 | B.1.1.7 | Male | ASYMPTOMATIC | 8.06 | Nasopharyngeal | 50 μL | Gatundu L5 |
| MKU_38 | B.1.1.7 | Female | ASYMPTOMATIC | 13.38 | Nasopharyngeal | 50 μL | St. Francis |
| MKU_39 | B.1 | Male | SYMPTOMATIC | 15.21 | Nasopharyngeal | 50 μL | St. Francis |
| MKU_40 | B.1.1.7 | Male | SYMPTOMATIC | 16.62 | Nasopharyngeal | 50 μL | St. Francis |
| MKU_43 | B.1.1.7 | Female | SYMPTOMATIC | 18.6 | Nasopharyngeal | 50 μL | Uhai Neema |
| MKU_45 | B.1.1.7 | Female | ASYMPTOMATIC | 11.53 | Nasopharyngeal | 50 μL | Uhai Neema |
| MKU_46 | B.1 | Male | SYMPTOMATIC | 8.06 | Nasopharyngeal | 50 μL | Gatundu L5 |
| MKU_47 | B.1.1.7 | Male | SYMPTOMATIC | 10.32 | Nasopharyngeal | 50 μL | Gatundu L5 |
| MKU_48 | B.1.1.7 | Female | SYMPTOMATIC | 16.27 | Nasopharyngeal | 50 μL | Gatundu L5 |
| MKU_49 | B.1 | Female | SYMPTOMATIC | 18.06 | Nasopharyngeal | 50 μL | Kiambu L5 |
| MKU_50 | B.1.617.2 | Male | SYMPTOMATIC | 14.28 | Nasopharyngeal | 50 μL | Kiambu L5 |
| MKU_51 | B.1.1 | Male | SYMPTOMATIC | 15.82 | Nasopharyngeal | 50 μL | Kiambu L5 |
| MKU_52 | B.1.617.2 | Female | ASYMPTOMATIC | 12.8 | Nasopharyngeal | 50 μL | Kiambu L5 |
| MKU_53 | B.1 | Male | ASYMPTOMATIC | 17.8 | Nasopharyngeal | 50 μL | Kiambu L5 |
| MKU_54 | B.1.617.2 | Female | ASYMPTOMATIC | 22.17 | Nasopharyngeal | 50 μL | Kiambu L5 |
| MKU_55 | B.1 | Female | ASYMPTOMATIC | 23.14 | Nasopharyngeal | 50 μL | Kiambu L5 |
| MKU_56 | B.1.617.2 | Female | ASYMPTOMATIC | 23.25 | Nasopharyngeal | 50 μL | Uhai Neema |
| MKU_58 | B.1.1 | Male | ASYMPTOMATIC | 15.5 | Nasopharyngeal | 50 μL | Uhai Neema |
| MKU_59 | B.1.1 | Male | SYMPTOMATIC | 28.66 | Nasopharyngeal | 50 μL | Uhai Neema |
| MKU_60 | B.1 | Male | ASYMPTOMATIC | 30.18 | Nasopharyngeal | 50 μL | Uhai Neema |
| MKU_61 | B.1.1.7 | Female | ASYMPTOMATIC | 30.86 | Nasopharyngeal | 50 μL | Uhai Neema |
| MKU_62 | B.1 | Male | SYMPTOMATIC | 31.59 | Nasopharyngeal | 50 μL | Uhai Neema |
| MKU_63 | B.1.1.7 | Female | ASYMPTOMATIC | 23.64 | Nasopharyngeal | 50 μL | St. Francis |
| MKU_64 | B.1.596 | Male | ASYMPTOMATIC | 23.71 | Nasopharyngeal | 50 μL | St. Francis |
| MKU_65 | A.2.5.1 | Male | ASYMPTOMATIC | 15.75 | Nasopharyngeal | 50 μL | St. Francis |
